# The survival of *Amblyomma sculptum* ticks upon blood-feeding depends on the expression of an inhibitor of apoptosis protein

**DOI:** 10.1186/s13071-023-05701-8

**Published:** 2023-03-10

**Authors:** Marcelly Nassar, Larissa A. Martins, Josiane Betim de Assis, Eliane Esteves, Anderson Sá-Nunes, Marcelo B. Labruna, Sirlei Daffre, Andrea C. Fogaça

**Affiliations:** 1 Departamento de Parasitologia, Instituto de Ciências Biomédicas, Universidade de São Paulo, São Paulo, SP Brazil; 2 National Institute of Allergy and Infectious Diseases, National Institutes of Health, Rocky Mountain Laboratories, Hamilton, MT USA; 3 Departamento de Imunologia, Instituto de Ciências Biomédicas, Universidade de São Paulo, São Paulo, SP Brazil; 4 Departamento de Medicina Veterinária Preventiva e Saúde Animal, Faculdade de Medicina Veterinária e Zootecnia, Universidade de São Paulo, São Paulo, SP Brazil

**Keywords:** Apoptosis, Tick, Caspase, IAP, Rickettsia

## Abstract

**Background:**

The tick *Amblyomma sculptum* is the major vector of *Rickettsia rickettsii*, the causative agent of the highly lethal Brazilian spotted fever. It has been shown that *R. rickettsii* inhibits apoptosis in both human endothelial cells and tick cells. Apoptosis is regulated by different factors, among which inhibitors of apoptosis proteins (IAPs) play a central role. In the study reported here, we selected an IAP of *A. sculptum* that has not yet been characterized to assess its role in cell death and to determine the effects of its gene silencing on tick fitness and *R. rickettsii* infection.

**Methods:**

An *A. sculptum* cell line (IBU/ASE-16) was treated with specific double-stranded RNA (dsRNA) for either IAP (dsIAP) or green fluorescent protein (dsGFP; as a control). The activity of caspase-3 and the exposure of phosphatidylserine were determined in both groups. In addition, unfed adult ticks, infected or not infected with *R. rickettsii*, were treated with either dsIAP or dsGFP and allowed to feed on noninfected rabbits. In parallel, noninfected ticks were allowed to feed on an *R. rickettsii*-infected rabbit. Ticks (infected or not with *R. rickettsii*) that remained unfed were used as a control.

**Results:**

Caspase-3 activity and the externalization of phosphatidylserine were significantly higher in IBU/ASE-16 cells treated with dsIAP than in those treated with dsGFP. The mortality rates of ticks in the dsIAP group were much higher than those in the dsGFP group when they were allowed to feed on rabbits, independent of the presence of *R. rickettsii*. Conversely, lower mortality rates were recorded in unfed ticks.

**Conclusions:**

Our results show that IAP negatively regulates apoptosis in *A. sculptum* cells. Moreover, IAP-silenced ticks experienced higher mortality rates following the acquisition of a blood meal, suggesting that feeding may trigger the activation of apoptosis in the absence of this physiological regulator. These findings indicate that IAP is a potential antigen for an anti-tick vaccine.

**Graphical Abstract:**

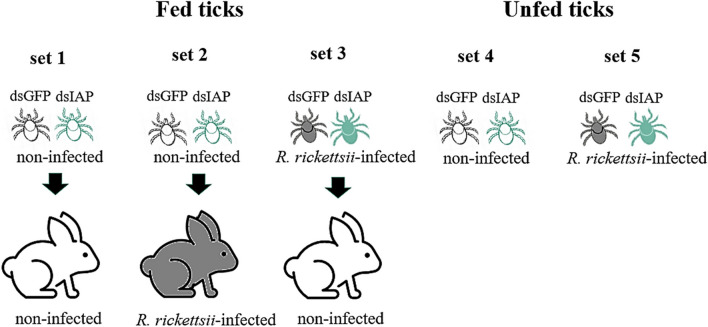

**Supplementary Information:**

The online version contains supplementary material available at 10.1186/s13071-023-05701-8.

## Background

The tick *Amblyomma sculptum*, a member of the *Amblyomma cajennense* species complex, is widely distributed throughout the Cerrado biome in South America, as well as in degraded areas of the Atlantic Forest in Brazil [1–3]. Although horses and capybaras are the preferred hosts of *A. sculptum*, this species infests several other wild and domestic hosts, including cattle and dogs [3, 4]. In Brazil, *A. sculptum* is the most frequent tick infesting humans [5], and, for humans, it is the main vector of the bacterium *Rickettsia rickettsii,* the causative agent of the highly lethal Brazilian spotted fever [6, 7].

In multicellular organisms, including ticks, apoptosis, which is a process of regulated cell death, is essential for development, homeostasis maintenance and immune response regulation [8]. Apoptosis also plays a direct role in the control of infections, as infected cells are eliminated, thereby preventing the dissemination of the infectious agent [8–10]. Apoptosis induced by either external or internal stimuli triggers the activation of effector caspases, such as caspase-3 and caspase-7, which coordinate a series of events that lead to cell death [11]. Inhibitors of apoptosis proteins (IAPs) are key regulators of apoptosis [12]. These proteins are characterized by the presence of one to three copies of the baculoviral IAP repeat (BIR) domain in the N-terminal region and a really interesting new gene (RING) domain in the C-terminal region. In mammals, IAPs can also exhibit a caspase recruitment domain (CARD) and an ubiquitin-associated (UBA) domain [12]. There are two types of BIR domains: type I and type II. The type II BIR domain binds to IAP binding motifs (IBM) present in caspases and IAP antagonists, while the type I BIR domain interacts with proteins involved in cell signaling pathways [12].

To ensure survival and proliferation within the host cell, some microorganisms can inhibit apoptosis [10, 13]. Previous studies have demonstrated that *R. rickettsii* is capable of inhibiting apoptosis in both human endothelial cells [14–16] and tick cells [17]. Infection with *R. rickettsii* was found to diminish caspase-3 activity in BME26 cells (derived from *Rhipicephalus microplus*) as well as in IBU/ASE-16 cells (derived from *A. sculptum*) [17]. Infection also reduced phosphatidylserine externalization in tick cells [17]. Interestingly, four coding sequences (CDS) of putative apoptosis regulators have been identified in the transcriptome of the *A. sculptum* midgut: one IAP (CDS Acaj-73060), one apoptotic inhibitor 5 (CDS Acaj-71920) and two apoptotic regulators of the BCL-2 (B-cell leukemia/lymphoma 2) family (CDSs Acaj-59320 and Acaj-73477) [18]. The gene expression of all of them, with the exception of CDS Acaj-71920, was upregulated by infection with *R. rickettsii* [18]. These results suggest that this bacterium may also be capable of inhibiting apoptosis in the tick midgut.

As IAPs play a central role in apoptosis regulation, directly interacting with caspases (such as caspase-3) and inhibiting their activity [12], we selected the IAP encoded by the CDS Acaj-73060 for functional characterization. We first administered double-stranded RNA (dsRNA) for either IAP or green fluorescent protein (GFP; used as a control) to IBU/ASE-16 cells and then evaluated caspase-3 activity and phosphatidylserine exposure in both groups of cells. To determine the effects of IAP knockdown on tick fitness and rickettsial infection, we administered dsIAP or dsGFP to either noninfected or *R. rickettsii*-infected unfed ticks and then allowed the ticks to feed on noninfected rabbits. A second group of noninfected ticks was fed on an *R. rickettsii*-infected rabbit. In addition, the gene expression of certain apoptosis regulators in the midgut of fed and unfed ticks was assessed.

## Methods

### Ethics statement

All procedures involving vertebrate animals were carried out according to Brazilian National Law (number 11794) and approved by the Institutional Animal Care and Use Committee from the Institute of Biomedical Sciences (CEUA number 1363060422) of the University of São Paulo (São Paulo, Brazil).

### Multiple sequence alignment and phylogenetic analysis

The amino acid sequence of the protein encoded by the CDS IAP Acaj-73060 (GenBank protein ID: JAT99717.1) was used as a query in searches against the National Center for Biotechnology Information (NCBI) Conserved Domain Database (CDD; http://www.ncbi.nlm.nih.gov/cdd) to identify conserved domains [19].

The amino acid sequence of the *A. sculptum* IAP was also used as a query in BLASTP searches against the Transcriptome Shotgun Assembly (tsa_nr; NCBI) database using the class Arachnida (taxid: 6854) as a filter. The protein sequence of a given tick species with the best match with *A. sculptum* (Additional file 1: Table S1) was selected as representative of a multiple sequence alignment (MSA) analysis using the MUSCLE (multiple sequence comparison by log-expectation) method [20] at the European Bioinformatics Institute (EMBL-EBI) website [21, 22]. Phylogenetic analysis of IAPs was conducted in MEGA11 software using the maximum likelihood method and the JTT matrix-based model [23].

### Tick cells

The embryonic cell line from *A. sculptum* IBU/ASE-16 [24] was cultured in commercial Leibovitz L-15 culture medium containing gentamicin sulfate and amphotericin (Vitrocell Embriolife, Campinas, SP, Brazil) and supplemented with 0.01% L-glutamine (Sigma‒Aldrich, St. Louis, MO, USA), as previously described [17].

### Ticks and* R. rickettsii*

Ticks were obtained from a laboratory colony of *A. sculptum* (Pedreira strain; São Paulo, Brazil). Larvae, nymphs and adults were fed on naïve rabbits (*Oryctolagus cuniculus*). Ticks in he off-host phases were maintained in an incubator at 25 °C and 90% relative humidity, as previously described [18]. For infection, larvae or adult ticks were allowed to feed on hosts infected with the Taiaçu strain of *R. rickettsii*, using previously described procedures [25]. The midgut of adult ticks was subsequently dissected as detailed in [18] and kept in RNAlater (Thermo Fisher Scientific, Waltham, MA, USA) at − 20 °C until nucleic acid extraction.

### DNA/RNA extraction and complementary DNA synthesis

IBU/ASE-16 cells and tick midgut samples were subjected to simultaneous isolation of genomic DNA and total RNA using the blackPREP Tick DNA/RNA Kit (Analytik Jena, Jena, Germany), according to the manufacturer’s specifications. The concentration of nucleic acids was determined using a spectrophotometer (NanoDrop-1000; Thermo Fisher Scientific).

For the synthesis of complementary DNA (cDNA), we treated the RNA with DNase and then used the treated RNA as a template for reverse transcription with the reverse transcriptase M-MLV (both enzymes from Thermo Fisher Scientific) according to the manufacturer’s protocol.

### dsRNA synthesis

Oligonucleotides for the synthesis of dsRNA for either the CDS IAP Acaj-73060 or GFP were designed and synthesized with a T7 promoter sequence coupled to the 5´ region (Additional file 2: Table S2). The cDNA previously synthesized from IBU/ASE-16 cell RNA and specific oligonucleotides for IAP were used to generate a 589-bp amplicon. As a control, a 300-bp amplicon for GFP was obtained using specific oligonucleotides and a plasmid containing a fragment of the GFP coding gene as a template.

Amplicons were separated by electrophoresis in an agarose gel stained with RED™ Gel (Uniscience Corp., Hialeah, FL, USA) and purified using the Wizard 46 SV Gel Clean-up System Kit (Promega, Madison, WI, USA). The purified cDNA was used as a template for dsRNA synthesis using the T7 Ribomax Express Kit System (Promega) according to the manufacturer’s recommendations.

### Caspase-3 assay

IBU/ASE-16 cells were transferred to 12.5-cm^2^ cell culture flasks containing 1.5 × 10^6^ cells each and, after 24 h at 30 °C, 10^13^ molecules of dsRNA for either IAP (dsIAP) or GFP (dsGFP) were added to the cells. After an additional 18 h of incubation, the cells were harvested and one-quarter of them was centrifuged at 400 *g* for 10 min at 4 °C and resuspended in RNAlater (Thermo Fisher Scientific) for nucleic acid extraction. The remaining cells were also centrifuged under the same conditions and then resuspended in lysis buffer [20 mM piperazine-N,N′-bis(2-ethanesulfonic acid) (PIPES), 100 mM NaCl, 2 mM ethylenediamine tetraacetic acid (EDTA), 0.1% 3-[(3-cholamidopropyl)dimethylammonio]-1-propanesulfonate hydrate (CHAPS), 10% sucrose, 0.1% Triton X-100, 1 mM phenylmethanesulfonyl fluoride (PMSF) and 2 μM pepstatin, pH 7.2]. The protein content in the resulting extracts was determined, and 50 μg of each sample was used to determine caspase-3 activity, assessed by the release of the aminomethyl coumarin (AMC) from the synthetic fluorogenic substrate Ac-DEVD-AMC, as previously described [17]. The value of arbitrary fluorescence units (UAF) obtained at time zero was subtracted from the value obtained after 60 min of reaction (∆UAF). Then, the relative activity of caspase-3 was calculated using the ratio of ∆UAF in the dsIAP group to ∆UAF of the reference condition (dsGFP). In experiments with the synthetic inhibitor Z-DEVD-Fmk, the relative activity of caspase-3 was calculated using the ratio of the ∆UAF in the presence of the inhibitor to ∆UAF in its absence in the dsIAP group. Six biological replicates of each condition were analyzed.

### Phosphatidylserine exposure analysis by flow cytometry

IBU/ASE-16 cells were transferred to 12.5-cm^2^ cell culture flasks containing 1.5 × 10^6^ cells each, and, after 24 h at 30 °C, the cells were treated with either dsIAP or dsGFP. After an additional 18 h of incubation, the cells were harvested and centrifuged at 400 *g* for 10 min at 4 °C, and one-quarter of the cells was resuspended in RNAlater (Thermo Fisher Scientific) for nucleic acid extraction. The remaining cells were washed with Hank’s balanced salt solution (HBSS) and then stained with Live/Dead Fixable Aqua (Thermo Fisher Scientific). After 15 min at 4 °C in the dark, the cells were washed, first with 2% phosphate-buffered saline and then with annexin-binding buffer (0.1 M HEPES, 1.4 M NaCl, 25 mM CaCl_2_), and then resuspended in 100 μl of annexin-binding buffer containing 5 μl of annexin V. After incubation for 10 min at room temperature with protection from light, an additional 100 μl of annexin-binding buffer was added to the cells. The cells were then analyzed in a FACSCanto II flow cytometer (BD Biosciences, San Jose, CA, USA) using FlowJo software, version 10.0.5 (BD Biosciences). Six biological replicates for each experimental group were evaluated.

### Administration of dsRNA to adult ticks

**Fig. 1:**
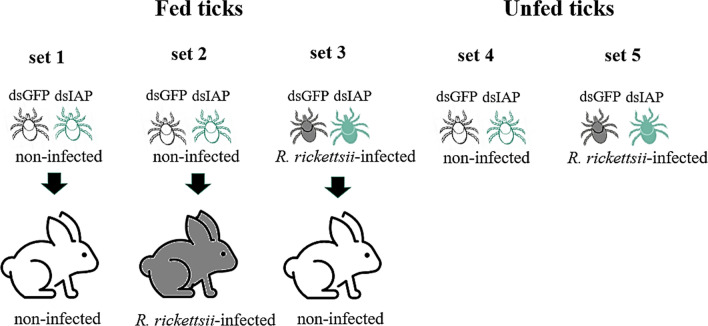
Schematic representation of the tick groups in the RNA interference experiments. Noninfected adult ticks injected with either dsIAP or dsGFP (control) were allowed to feed for 7 days on either a noninfected rabbit (set 1) or a *R. rickettsii*-infected rabbit (set 2). *Rickettsia rickettsii*-infected ticks were also injected with either dsIAP or dsGFP and allowed to feed on a noninfected rabbit (set 3). In parallel, uninfected (set 4) or *R. rickettsii*-infected (set 5) ticks that received dsRNAs for IAP or GFP were maintained unfed. dsGFP, double-stranded (ds) RNA for green fluorescent protein (GFP); dsIAP, double-stranded (ds) RNA for inhibitor of apoptosis protein (IAP)

Adult *A. sculptum* ticks were injected with 10^11^ molecules of either dsIAP or dsGFP solubilized in 69 nl of water, as described previously [26]. After 24 h at 30 °C, ticks were either allowed to feed on rabbits (sets 1, 2 and 3) or they remained without feeding (sets 4 and 5) (Fig. 1). Set 1 corresponds to uninfected ticks fed on an uninfected rabbit; set 2 corresponds to uninfected ticks fed on an *R. rickettsii*-infected rabbit; and set 3 corresponds to *R. rickettsii*-infected ticks (infected as larvae by feeding on an infected rabbit) fed on an uninfected rabbit. Sets 4 and 5 correspond to unfed ticks, either uninfected (set 4) or infected (set 5) with *R. rickettsii*. In sets 4A and 5A, tick viability was evaluated for 8 days after dsRNA administration, while in sets 4B and 5B, tick viability was evaluated until 30 days post-injection. Twenty-five ticks (each representing one biological replicate) were used for each group (dsIAP and dsGFP) of sets 1, 2, and 3, while 10 biological replicates were used for each group of sets 4 (A and B) and 5 (A and B).

### Quantitative PCR preceded by reverse transcription

Specific oligonucleotides for selected genes were designed using Primer3 [27] (Additional file 2: Table S2) and used to assess their expression by quantitative PCR preceded by reverse transcription (RT‒qPCR). Reactions were carried out using Maxima SYBR Green/ROX qPCR MasterMix and a StepOnePlus™ thermocycler with the help of the program StepOne v2.3 (reagent, equipment and software from Thermo Fischer Scientific). The amount of cDNA in the samples was normalized to the expression of the gene encoding the S3A ribosomal protein of *A. sculptum* [18].

In the RNA interference (RNAi) experiments, the relative mRNA levels of the CDS Acaj-73060 in the dsIAP group in relation to that in the dsGFP group in IBU/ASE-16 cells and in the midgut of *A. sculptum* ticks were calculated by the 2^−∆∆Ct^ method, according to the interpretation proposed by [28]. The percentage of gene silencing was obtained considering the IAP expression level in the control (dsGFP) to be 100%. Six biological replicates of each condition were analyzed.

The expression level of apoptosis regulators [IAP (CDS Acaj-73060), apoptotic inhibitor 5 (CDS Acaj-71920) and apoptotic regulators of the BCL-2 family (CDSs Acaj-59320 and Acaj-73477)] in the midgut of *A. sculptum* fed for 72 h in relation to unfed ticks was also determined by the 2^−∆∆Ct^ method. Six biological replicates of each condition were analyzed.

### Statistical analyses

Data were statistically analyzed using the Mann‒Whitney test with GraphPad Prism software (GraphPad Software Inc., version 6.0, San Jose, CA, USA). The differences between the groups were considered to be significant when *P* ˂ 0.05.

## Results

### Phylogeny of IAP family mirrors tick taxonomy

The CDD analysis of the amino acid sequence of *A. sculptum* IAP showed that it contains three type II BIR domains in its N-terminal region and a RING domain in its C-terminal region. These domains are conserved in the IAPs of other tick species, including the XIAP of *Ixodes scapularis* [29], as revealed by MSA analysis (Additional file 3: Figure S1A). The phylogenetic tree resembled the Ixodidae and Argasidae taxonomic clades, with species within the same genus clustering together (Additional file 3: Figure S1B).

### Effects of IAP knockdown on caspase-3 activity and phosphatidylserine exposure in IBU/ASE-16 cells

**Fig. 2:**
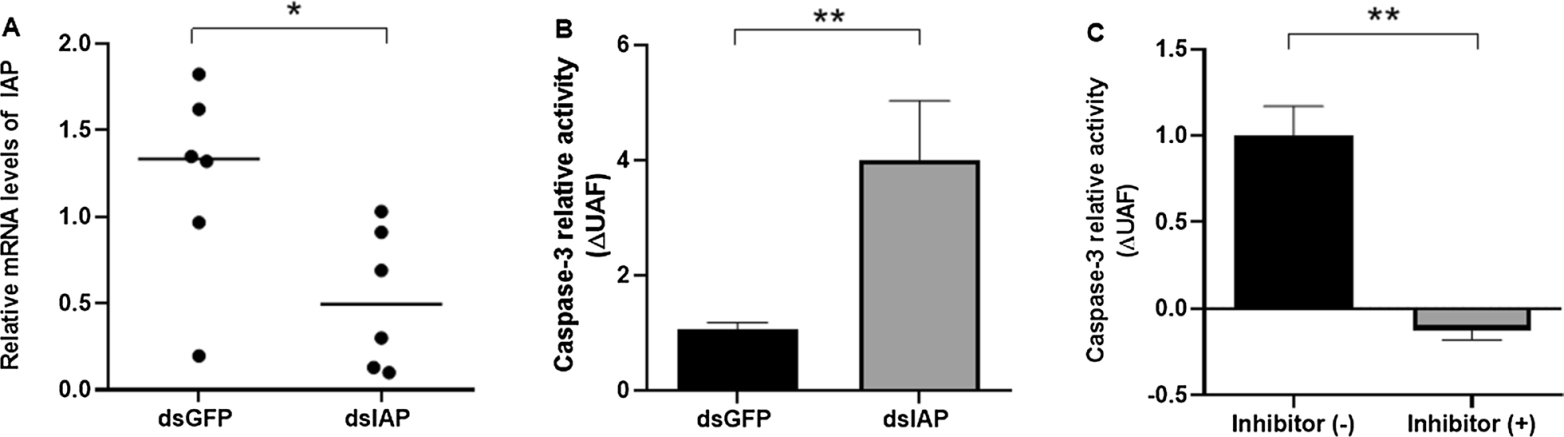
Analysis of IAP gene expression and caspase-3 activity in IBU/ASE-16 cells upon IAP silencing. **a** IAP Acaj-73060 gene expression in cells treated with either dsIAP or dsGFP was determined by RT-qPCR. The relative mRNA levels of IAP the dsIAP group in relation to that in the dsGFP group were calculated by the 2^-ΔΔCt^ method.  **b** Caspase-3 activity in dsIAP- or dsGFP-treated cells was determined by measuring the release of the aminomethyl coumarin (AMC) fluorescent cleavage product from the synthetic fluorogenic substrate Ac-DEVD-AMC. The relative activity of caspase-3 [in units of arbitrary fluorescence (UAF)] represents the ratio of ΔUAF (UAF_60min_ − UAF_0min_) of the dsIAP group to the control (dsGFP) group. **c** Caspase-3 activity in cells treated with dsIAP in the presence or the absence of the synthetic inhibitor Z-DEVD-Fmk was determined using the same procedure. The relative activity of caspase-3 (UAF) represents the ratio of ΔUAF (UAF_60min_ – UAF_0min_) in the presence of Z-DEVD-Fmk to ΔUAF in the control (absence of the inhibitor). Error bars: ± standard deviation (SD) of 6 measurements (*n* = 6). Asterisks indicate a significant difference at *P < 0.05 and **P < 0.01

To determine the effects of IAP silencing on the activity of caspase-3, we incubated IBU/ASE-16 cells with either dsIAP or dsGFP (Fig. 2). IAP expression was significantly lower in cells treated with dsIAP than in the control cells, with a gene silencing of 57% (*P* = 0.041; Fig. 2a). On the other hand, caspase-3 activity was significantly higher in cells treated with dsIAP than in cells treated with dsGFP (*P* = 0.0022; Fig. 2b). To determine whether the caspase-3 activity was specific, we performed an enzymatic assay with its synthetic inhibitor Z-DEVD-Fmk and found that caspase-3 activity in the dsIAP group was significantly higher in the absence of its specific inhibitor than in its presence (*P* = 0.0022; Fig. 2c).

**Fig. 3:**
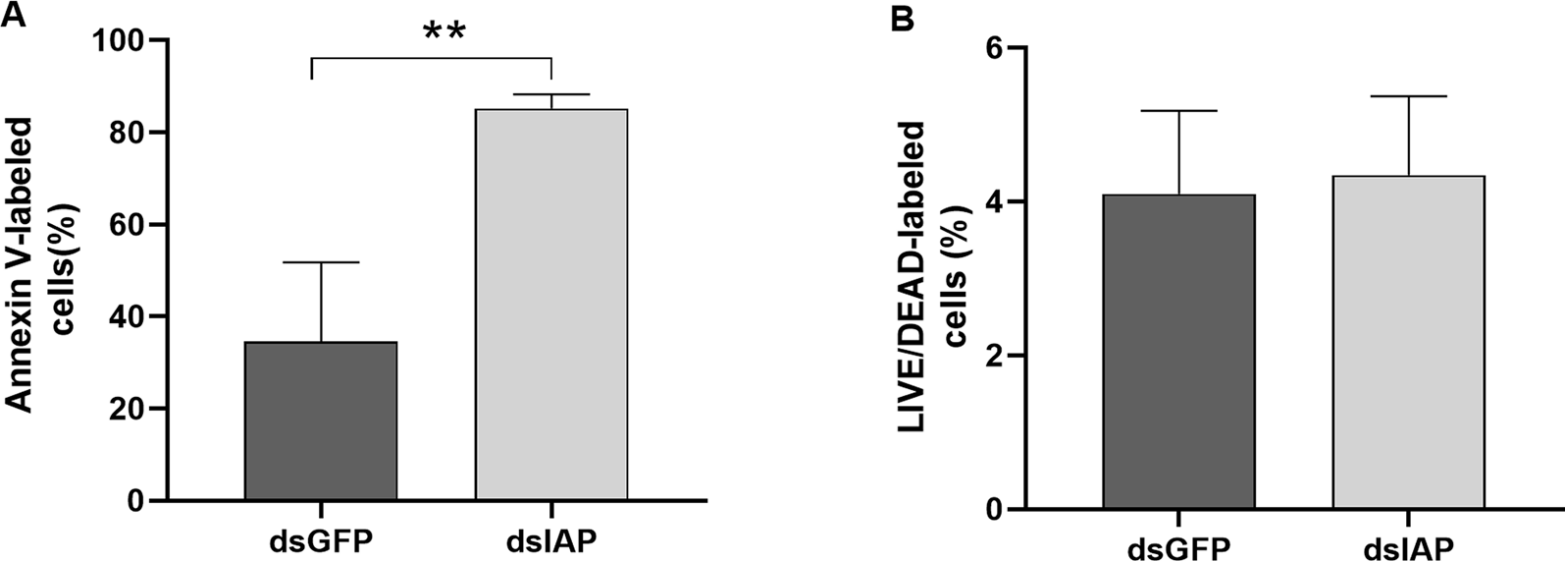
Flow cytometry analysis of phosphatidylserine exposure and viability of IBU/ASE-16 cells upon IAP silencing. Percentage (%) of cells labeled with annexin V **a** and with Live/Dead **b**. Asterisks indicate a significant difference at **P < 0.01

To evaluate phosphatidylserine exposure and cell viability, IBU/ASE-16 cells were treated with either dsIAP or dsGFP, labeled with annexin-V and with Live/Dead and analyzed by flow cytometry. The dsIAP group showed a significantly higher percentage of annexin V-labeled cells than the dsGFP-treated group (*P* = 0.0079; Fig. 3a). In terms of cell viability, there was no difference between the dsIAP and dsGFP groups (*P* = 0.8413; Fig. 3B).

### IAP knockdown results in mortality of* A. sculptum* following blood-feeding, independent of* R. rickettsii* infection

To evaluate whether IAP silencing could interfere with tick fitness and/or rickettsial colonization, we treated adult *A. sculptum* ticks with either dsIAP or dsGFP. Five different conditions were analyzed (Fig. 1).

High mortality rates were recorded in the dsIAP group in comparison to the dsGFP (control) group when ticks were allowed to feed on rabbits, independent of the presence of *R. rickettsii* in the host or the ticks (Table 1). In set 1, all 25 ticks from the dsIAP group died (100% mortality) after 3 days of feeding onset, in contrast to only one dead tick in the dsGFP group (4% mortality). In set 2, 23 of 25 ticks (92% mortality) in the dsIAP group died after feeding (Additional file 4: Figure S2A), while in the control group, only two specimens (8% mortality) died. In set 3, 24 ticks from the dsIAP group (96% mortality) and seven (28% mortality) from the dsGFP group died.

**Table 1:** Tick mortality rates in the different experimental settings of the study

Label for experimental setting	Conditions of experimental setting	Mortality rate	
		dsIAP	dsGFP
Set 1	Uninfected ticks feeding on an uninfected rabbit	100% (25/25)	4% (1/25)
Set 2	Uninfected ticks feeding on an *R. rickettsii-*infected rabbit	92% (23/25)	8% (2/25)
Set 3	*R. rickettsii-*infected ticks feeding on an uninfected rabbit	96% (24/25)	28% (7/25)
Set 4A	Unfed uninfected ticks (8 days after dsRNA administration)	0% (0/10)	0% (0/10)
Set 4B	Unfed uninfected ticks (30 days after dsRNA administration)	0% (0/10)	0% (0/10)
Set 5A	Unfed *R. rickettsii-*infected ticks (8 days after dsRNA administration)	60% (6/10)	40% (4/10)
Set 5B	Unfed *R. rickettsii-*infected ticks (30 days after dsRNA administration)	40% (4/10)	20% (2/10)

dsGFP Double-stranded (ds) RNA for green fluorescent protein (GFP), dsIAP double-stranded (ds) RNA for inhibitor of apoptosis protein (IAP)

In contrast to the high mortality of ticks in the dsIAP groups of sets 1, 2 and 3, which were allowed to feed on rabbits, much lower mortality rates were recorded in unfed ticks in sets 4 and 5 (Table 1). Indeed, all of the ticks in the dsIAP and dsGFP groups of set 4 remained alive even 30 days after dsRNA administration (Additional file 4: Figures S2 and, C, respectively). In set 5, tick mortality rates in the dsIAP group at 8 (set 5A) and 30 days (set 5B) after dsRNA administration were 60% and 40%, respectively. The highest mortality rates for a dsGFP group were detected in ticks of sets 3 and 5 (Table 1).

**Fig. 4:**
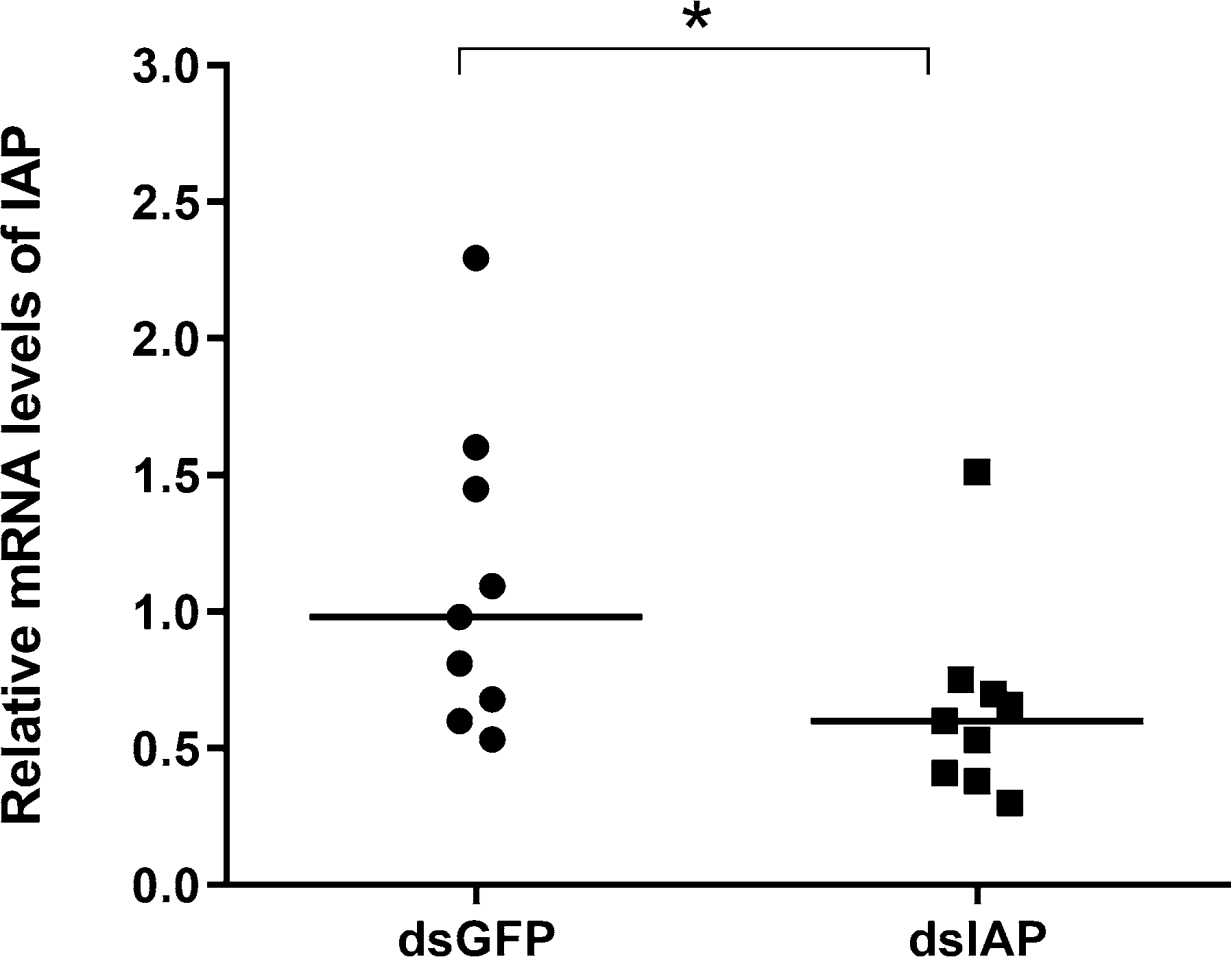
Analysis of IAP gene expression in the midgut of ticks by RT-qPCR. The relative mRNA levels of IAP in the midgut of ticks from the dsIAP group in relation to that in the midgut of ticks from the dsGFP group of set 4A (unfed and uninfected ticks) were calculated by the 2^-ΔΔCt^ method. The asterisk indicates a significant difference at *P < 0.05

Due to the high mortality rates of ticks in sets 1, 2 and 3, it was not possible to determine the gene expression of IAP in ticks treated with dsIAP in relation to those treated with dsGFP. Thus, IAP gene expression was determined in ticks of set 4A, in which all ticks survived. IAP gene expression was significantly lower in the dsIAP group than in the control group, with silencing of 42% (*P* = 0.037; Fig. 4).

### Blood-feeding downregulates the gene expression of apoptosis regulators in the midgut of* A. sculptum*

**Fig. 5:**
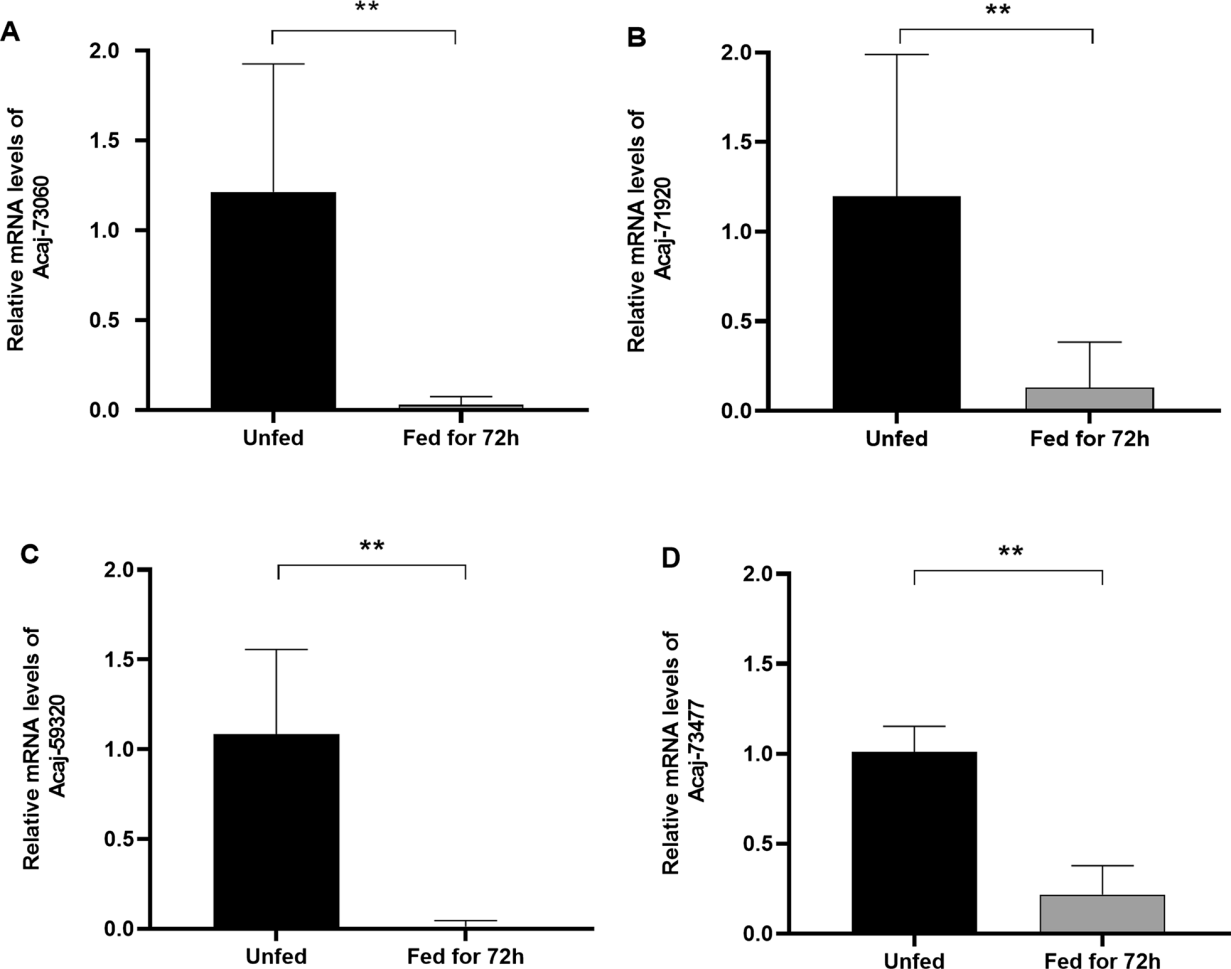
Gene expression of apoptosis modulators in the midgut of *A. sculptum* ticks. The relative expression of IAP (Acaj-73060; **a**), apoptotic inhibitor 5 (Acaj-71920; **b**) and apoptotic regulators of the BCL-2 family (Acaj-59320 **c** and Acaj-73477 **d**) in the midgut of ticks fed for 72 h in relation to that in unfed ticks were calculated by the 2^-ΔΔCt^ method. Asterisks indicate a significant difference at **P < 0.01

The gene expression of CDSs Acaj-73060 (IAP), Acaj-71920 (apoptotic inhibitor 5) and Acaj-59320 and Acaj-73477 (apoptotic regulators of the BCL-2 family) in ticks fed for 72 h in relation to unfed ticks was assessed. The results showed that the transcript levels of IAP Acaj-73060 were significantly lower in fed ticks than in unfed ticks (*P* = 0.0025; Fig. 5a). Figures 5b–d show a similar downregulation of Acaj-71920 (*P* = 0.0051), Acaj-59320 (*P* = 0.0025) and Acaj-73477 (*P* = 0.0025), respectively, in fed ticks.

## Discussion

*Rickettsia rickettsii* has been shown to be able to inhibit apoptosis in human endothelial cells [14–16] in a nuclear factor-kappa B (NF-κB)-dependent manner [16]. Accordingly, a previous study conducted by our group showed that *R. rickettsii* inhibits apoptosis in embryonic cells of the ticks *R. microplus* (BME26) and *A. sculptum* (IBU/ASE-16) [17]. It should be noted that *R. rickettsii* growth is faster in BME26 cells treated with a caspase-3 inhibitor than in control (untreated) cells while, in contrast, rickettsial growth is much slower in cells treated with staurosporine, a classic apoptosis activator. These results indicate that the inhibition of apoptosis is important for *R. rickettsii* survival and growth in tick cells [17]. Interestingly, RNA-seq analyses [18] revealed that negative modulators of apoptosis, including one IAP (CDS Acaj-73060), were upregulated in the midgut of infected *A. sculptum*, suggesting that *R. rickettsii* may also control apoptosis in vivo.

Our in silico analysis of the IAP Acaj-73060 amino acid sequence showed that it possesses three type II BIR domains in the N-terminal region (Additional file 3: Figure S1A). As type II BIR domains inhibit the activity of caspases through direct interactions with these enzymes [12], we determined the effects of IAP silencing on the caspase-3 activity of IBU/ASE-16 cells (Fig. 2b). The results showed that the enzymatic activity of caspase-3 was significantly higher in IAP-silenced cells than in control cells (cells treated with dsGFP), demonstrating that IAP Acaj-73060 plays a role in apoptosis inhibition. Importantly, caspase-3 activity in dsIAP-treated cells was completely abolished by incubation with the caspase-3 inhibitor Z-DEVD-Fmk (Fig. 2c), supporting its specificity. In addition, IAP-silencing significantly diminished the exposure of phosphatidylserine in IBU/ASE-16 cells (Fig. 3), supporting the hypothesis that this protein can negatively regulate apoptosis.

The mortality rates of IAP-silenced ticks allowed to feed on rabbits were much higher than those of nonsilenced ticks (treated with dsGFP), independent of the presence of *R. rickettsii* within the host blood or the tick tissues (Table 1). On the other hand, the mortality rates in unfed ticks of the dsIAP group were much lower (Table 1). Remarkably, all noninfected unfed ticks from the dsGFP and dsIAP groups remained alive even at 30 days after injection (Table 1, set 4B), while mortality rates of 40% and 20% in these two groups, respectively (Table 1, set 5B), were obtained for *R. rickettsii*-infected ticks. Indeed, the highest mortality rates of ticks that received dsGFP were obtained in ticks from set 3 and sets 5A and 5B (ticks infected as larvae with *R. rickettsii*). A number of published studies have reported that *R. rickettsii*-infected ticks present higher mortality rates than uninfected ticks, suggesting a possible pathogenic effect of this bacterium on its vectors in the USA and Brazil [30–32]. Therefore, it is possible that infected ticks may be more susceptible to intrathoracic injection for the administration of dsRNA.

The detrimental effect of dsIAP administration followed by feeding suggests that the acquisition of the blood meal might activate apoptosis, causing tick death. Therefore, the gene expression of negative regulators of apoptosis in fed ticks in comparison to unfed ticks was evaluated. Our analyses showed that the expression of all CDSs, including the CDS of IAP Acaj-73060, was downregulated in fed ticks (Fig. 5). In addition to the downregulation of negative modulators of apoptosis, acquiring a blood meal might also induce the production of reactive oxygen species (ROS) in the tick midgut, which in turn may activate apoptosis. Indeed, it is known that an imbalance in redox metabolism and an increase in ROS concentration within the cell can activate apoptosis [33]. Moreover, previous studies by our research group showed that CDSs of antioxidant proteins from *R. rickettsii* are induced in the midgut of fed ticks, suggesting that feeding promotes the formation of a pro-oxidant environment [25, 34].

Due to the high mortality rates of blood-fed ticks treated with dsIAP, it was not possible to analyze the role of IAP in rickettsial proliferation in ticks from sets 2 and 3. However, the knockdown of XIAP from *I. scapularis*, which is similar to IAP Acaj-73060 from *A. sculptum* (Additional file 3: Figure S1A), increased the colonization of the tick midgut by *Anaplasma phagocytophilum* [29]. To date, the XIAP of *I. scapularis* has only been shown to be involved in the IMD pathway, but not in apoptosis. Upon microbial activation, XIAP, together with the heterodimer E2 conjugating enzyme complex Bendless:UEV1a, binds and ubiquitylates its p47 substrate in a K63-dependent manner. Subsequently, ubiquitylated p47 binds to Kenny (a regulatory subunit of the NF-κB kinase complex), inducing the phosphorylation of Relish, the transcription factor of this immune pathway, which is cleaved and translocated to the nucleus [35]. This E3-ubiquitin ligase activity of XIAP is conferred by its RING domain. Although the IAP Acaj-73060 of *A. sculptum* also contains a RING domain, its E3-ubiquitin ligase activity was not evaluated in the present study. Intriguingly, *A. phagocytophilum* has been previously described to inhibit apoptosis in the midgut and in the salivary glands of *I. scapularis* [36]. In the salivary glands, bacterial infection inhibits the intrinsic pathway of apoptosis by downregulating porin expression, which results in the inhibition of cytochrome* c* release [36]. XIAP silencing negatively affected *I. scapularis* fitness, causing a decrease in female engorgement [36]. However, none of these studies showed the high tick mortality rates recorded in the present study.

## Conclusions

Our findings demonstrate that *A. sculptum* IAP negatively regulates apoptosis in IBU/ASE-16 cells. In addition, the gene silencing of *A. sculptum* IAP Acaj-73060, followed by the acquisition of a blood meal, caused a high mortality of ticks, suggesting that apoptosis regulation is essential to successful tick feeding. These results identify this protein as a target for the development of an anti-tick vaccine. Additional studies are needed to determine how the acquisition of the blood meal leads to the activation of apoptosis in the *A. sculptum* midgut.

## Supplementary Information

**Additional file 1: Table S1. **Accession numbers of IAP sequences used to perform the multiple sequence alignment and generate a phylogenetic tree are displayed in Figure 2.

**Additional file 2: Table S2. **Specific oligonucleotides used for dsRNA synthesis and RT‒qPCR analyses.

**Additional file 3: Figure S1**. **A** Multiple sequence alignment of amino acid sequences was performed using the MUSCLE method. BIR domains are shown in gray boxes, and RING domain is shown in bold letters. **B** Phylogenetic tree of tick IAPs. The tree was constructed with IAP amino acid sequences from 15 tick species (accession numbers available in Additional file 1: Table S1) using the maximum likelihood method. The bar scale at the bottom indicates 10% amino acid divergence.

**Additional file 4: Figure S2**. **A** Dead ticks from the dsIAP group of set 2 (uninfected ticks fed for 8 days on an *R. rickettsii*-infected rabbit). **B** Live ticks from the dsIAP group of set 4B (uninfected and unfed ticks 30 days postinjection).** C** Live ticks from the dsGFP group of set 4B (uninfected and unfed ticks 30 days postinjection).

## Data Availability

The authors declare that all data supporting the findings of this study are available within the article and its Additional files.

## Abbreviations

BCL-2: B-cell leukemia/lymphoma 2
BIR: Baculoviral IAP repeat
CDD: Conserved Domain Database
cDNA: Complementary DNA
CDS: Coding sequence
dsRNA: Double-stranded RNA
GFP: Green fluorescent protein
IAPs: Inhibitors of apoptosis proteins
mRNA: messenger RNA
RING: Really interesting new gene
RT-qPCR: quantitative PCR preceded by reverse transcription
UAF: Arbitrary fluorescence units

## References

[1] Nava S, Beati L, Labruna M, Cáceres A, Mangold A, Guglielmone A (2014). Reassessment of the taxonomic status of *Amblyomma cajennense* () with the description of three new species, Amblyomma tonelliae n. sp., Amblyomma interandinum n. sp. and Amblyomma patinoi n. sp., and reinstatement of Amblyomma mixtum, and *Amblyomma sculptum* (*Ixodida: Ixodidae*). Ticks Tick Borne Dis.

[2] Martins TF, Barbieri AR, Costa FB, Terassini FA, Camargo LM, Peterka CR, et al. Geographical distribution of *Amblyomma cajennense* (sensu lato) ticks (Parasitiformes: *Ixodidae*) in Brazil, with description of the nymph of *A. cajennense* (sensu stricto). Parasit Vectors. 2016;9:186. 10.1186/s13071-016-1460-2. 10.1186/s13071-016-1460-2 PMC4818509 27036324

[3] Krawczak FS, Nieri-Bastos FA, Nunes FP, Soares JF, Moraes-Filho J, Labruna MB. *Rickettsial* infection in *Amblyomma cajennense* ticks and capybaras (*Hydrochoerus hydrochaeris*) in a Brazilian spotted fever-endemic area. Parasit Vectors. 2014;7:7. 10.1186/1756-3305-7-7. 10.1186/1756-3305-7-7 PMC3892071 24387674

[4] de Siqueira SM, da Costa Maia R, do Nascimento Ramos V, da Silva Rodrigues V, Szabó MPJ (2021). Rhipicephalus microplus and *Amblyomma sculptum* (*Ixodidae*) infestation of Nellore cattle (Bos taurus indicus) in a farm of the Brazilian Cerrado: seasonality and infestation patterns. Exp Appl Acarol.

[5] Nogueira BCF, Campos AK, Muñoz-Leal S, Pinter A, Martins TF (2022). Soft and hard ticks (Parasitiformes: *Ixodida*) on humans: a review of Brazilian biomes and the impact of environmental change. Acta Trop.

[6] Labruna MB (2009). Ecology of rickettsia in South America. Ann N Y Acad Sci.

[7] Dantas-Torres F (2007). Rocky mountain spotted fever. Lancet Infect Dis.

[8] Galluzzi L, Vitale I, Aaronson SA, Abrams JM, Adam D, Agostinis P (2018). Molecular mechanisms of cell death: recommendations of the nomenclature committee on cell death 2018. Cell Death Differ.

[9] Cooper DM (2011). Death for survival: what do we know about innate immunity and cell death in insects ? heart and lung. Invertebr Surviv J.

[10] Ashida H, Mimuro H, Ogawa M, Kobayashi T, Sanada T, Kim M (2011). Cell death and infection: a double-edged sword for host and pathogen survival. J Cell Biol.

[11] Elmore S (2007). Apoptosis: a review of programmed cell death. Toxicol Pathol.

[12] Berthelet J, Dubrez L (2013). Regulation of apoptosis by inhibitors of apoptosis (IAPs). Cells.

[13] Rudel T, Kepp O, Kozjak-Pavlovic V (2010). Interactions between bacterial pathogens and mitochondrial cell death pathways. Nat Rev Microbiol.

[14] Joshi SG, Francis CW, Silverman DJ, Sahni SK (2003). Nuclear factor κB protects against host cell apoptosis during *Rickettsia rickettsii* infection by inhibiting activation of apical and effector caspases and maintaining mitochondrial integrity. Infect Immun.

[15] Joshi S, Francis C, Silverman D, Sahni S (2004). NF-kappaB activation suppresses host cell apoptosis during *Rickettsia rickettsii* infection via regulatory effects on intracellular localization or levels of apoptogenic and anti-apoptotic proteins. FEMS Microbiol Lett.

[16] Clifton DR, Goss RA, Sahni SK, van Antwerp D, Baggs RB, Marder VJ, Silverman DJ, Sporn LA (1998). NF- B-dependent inhibition of apoptosis is essential for host cell survival during *Rickettsia rickettsii* infection. Proc Natl Acad Sci USA.

[17] Martins LA, Palmisano G, Cortez M, Kawahara R, de Freitas Balanco JM, Fujita A (2020). The intracellular bacterium *Rickettsia rickettsii* exerts an inhibitory effect on the apoptosis of tick cells. Parasit Vectors.

[18] Martins L, Galletti M, Ribeiro JM, Fujita A, Costa FB, Labruna MB, et al. The distinct transcriptional response of the midgut of *Amblyomma sculptum* and amblyomma aureolatum ticks to *Rickettsia rickettsii* correlates to their differences in susceptibility to infection. Front Cell Infect Microbiol. 2017;7. 10.3389/fcimb.2017.00129. 10.3389/fcimb.2017.00129 PMC5409265 28503490

[19] Marchler-Bauer A, Derbyshire MK, Gonzales NR, Lu S, Chitsaz F, Geer LY, et al. CDD: NCBI’s conserved domain database. Nucleic Acids Res. 2015;43:D222–6. 10.1093/nar/gku1221. 10.1093/nar/gku1221 PMC4383992 25414356

[20] Edgar RC (2004). MUSCLE: a multiple sequence alignment method with reduced time and space complexity. BMC Bioinformatics.

[21] Li W, Cowley A, Uludag M, Gur T, McWilliam H, Squizzato S, et al. Analysis tool web services from the EMBL-EBI. Nucleic Acids Res. 2013;41:W597–600. 10.1093/nar/gkt376. 10.1093/nar/gkt376 PMC3692137 23671338

[22] Li W, Cowley A, Uludag M, Gur T, McWilliam H, Squizzato S (2015). The EMBL-EBI bioinformatics web and programmatic tools framework. Nucleic Acids Res.

[23] Kumar S, Stecher G, Li M, Knyaz C, Tamura K (2018). MEGA X: molecular evolutionary genetics analysis across computing platforms. Mol Biol Evol.

[24] Moraes AC. Establishment and characterization of embryonic cells of* Amblyomma sculptum* Berlese (Acari: Ixodidae). 2016. São Paulo: Biblioteca Digital de Teses e Dissertações da Universidade de São Paulo. https://www.teses.usp.br/teses/disponiveis/87/87131/tde-04122015-152642. Accessed 01 Nov 2022.

[25] Galletti MF, Fujita A, Nishiyama MY, Malossi CD, Pinter A, Soares JF (2013). Natural blood feeding and temperature shift modulate the global transcriptional profile of *Rickettsia rickettsii* infecting its tick vector. PLoS ONE.

[26] Martins LA, Malossi CD, Galletti MFBM, Ribeiro JM, Fujita A, Esteves E (2019). The transcriptome of the salivary glands of *Amblyomma aureolatum* reveals the antimicrobial peptide microplusin as an important factor for the tick protection against *Rickettsia rickettsii* infection. Front Physiol.

[27] Rozen S, Skaletsky H (2000). Primer3 on the WWW for general users and for biologist programmers. Methods Mol Biol.

[28] Livak K, Schmittgen T (2001). Analysis of relative gene expression data using real-time quantitative PCR and the 2(-Delta Delta C(T) method. Methods.

[29] Severo MS, Choy A, Stephens KD, Sakhon OS, Chen G, Chung DW, et al. The E3 ubiquitin ligase XIAP restricts *Anaplasma phagocytophilum* colonization of *Ixodes scapularis* ticks. J Infect Dis. 2013;208:1830–40. 10.1093/infdis/jit380. 10.1093/infdis/jit380 PMC3814841 23901084

[30] Niebylski ML, Peacock MG, Schwan TG (1999). Lethal effect of *Rickettsia rickettsii* on its tick vector (*Dermacentor andersoni*). Appl Environ Microbiol.

[31] Labruna MB, Ogrzewalska M, Soares JF, Martins TF, Soares HS, Moraes-Filho J (2011). Experimental Infection of *Amblyomma aureolatum* ticks with *Rickettsia rickettsii*. Emerg Infect Dis.

[32] Soares JF, Soares HS, Barbieri AM, Labruna MB (2012). Experimental infection of the tick *Amblyomma cajennense*, cayenne tick, with *Rickettsia rickettsii*, the agent of rocky mountain spotted fever. Med Vet Entomol.

[33] Redza-Dutordoir M, Averill-Bates DA (2016). Activation of apoptosis signalling pathways by reactive oxygen species. Biochim Biophys Acta.

[34] Galletti MF, Fujita A, Rosa RD, Martins LA, Soares HS, Labruna MB (2016). Virulence genes of Rickettsia rickettsii are differentially modulated by either temperature upshift or blood-feeding in tick midgut and salivary glands. Parasit Vectors.

[35] Shaw DK, Wang X, Brown LJ, Chávez AS, Reif KE, Smith AA, et al. Infection-derived lipids elicit an immune deficiency circuit in arthropods. Nat Commun. 2017;8:14401. 10.1038/ncomms14401. 10.1038/ncomms14401 PMC5316886 28195158

[36] Ayllón N, Villar M, Galindo RC, Kocan KM, Šíma R, López JA (2015). Systems biology of tissue-specific response to *Anaplasma phagocytophilum* reveals differentiated apoptosis in the tick vector *Ixodes scapularis*. PLoS Genet.

