# Supplementary material for: The survival of Amblyomma sculptum ticks upon blood-feeding depends on the expression of an inhibitor of apoptosis protein
Source: Parasit Vectors. 2023 Mar 10;16:96. doi: 10.1186/s13071-023-05701-8 (PMC10007823; doi:10.1186/s13071-023-05701-8)
Supplement: Supplementary file 1 — Additional file 1: Table S1. Accession numbers of IAP sequences used to perform the multiple sequence alignment and generate a phylogenetic tree are displayed in Figure 2. [file 13071_2023_5701_MOESM1_ESM.pdf]

**Additional file 1: Table S1.** Accession numbers of IAP amino acid sequences used to perform multiple sequence alignment and generate a phylogenetic tree displayed in Figure 2.

| **Ticks species** | **Accession number** |
|---|---|
| *Amblyomma sculptum* | JAT99717.1 |
| *Amblyomma aureolatum* | JAT95460.1 |
| *Amblyomma maculatum* | MCL6769568.1 |
| *Amblyomma triste* | JAC33924.1 |
| *Dermacentor reticulatus* | MBD2901002.1 |
| *Hyalomma excavatum* | JAP65768.1 |
| *Ixodes ricinus* | AR90216.1 |
| *Ixodes scapularis* | MOY37746.1 |
| *Ornithodoros erraticus* | MBZ3995776.1 |
| *Ornithodoros moubata* | MBZ3985256.1 |
| *Ornithodoros turicata* | MBY04351.1 |
| *Rhipicephalus appendiculatus* | JAP78245.1 |
| *Rhipicephalus microplus* | NOV33869.1 |
| *Rhipicephalus pulchellus* | JAA59147.1 |
| *Rhipicephalus zambeziensis* | MAA20231.1 |
