# Supplementary material for: The survival of Amblyomma sculptum ticks upon blood-feeding depends on the expression of an inhibitor of apoptosis protein
Source: Parasit Vectors. 2023 Mar 10;16:96. doi: 10.1186/s13071-023-05701-8 (PMC10007823; doi:10.1186/s13071-023-05701-8)
Supplement: Supplementary file 2 — Additional file 2: Table S2. Specific oligonucleotides used for dsRNA synthesis and RT‒qPCR analyses. [file 13071_2023_5701_MOESM2_ESM.pdf]

Additional file 2: Table S2. Specific oligonucleotides (sense and antisense) used for dsRNA synthesis and RT-qPCR analyses.

CDS	Annotation	Sense (sequence 5´ → 3´)	Antisense (sequence 5´ → 3´)
	dsGFP	*TAATACGACTCACTATAGG*TTCACTGGAGTTGTCCCAAT	*TAATACGACTCACTATAGG*CTTGTAGTTCCCGTCATCTT
	dsIAP	*TAATACGACTCACTATAGG*CTTGAAATTGGGGACTGGAA	*TAATACGACTCACTATAGG*TACCATTCGCTCCGATGAGC
	Ribosomal protein S3A	TACCTGCTGCGAATGTTCTG	TTCTTCCTGATGAGGCGA
Acaj-73060	IAP	TGTACGGGTCGCCTCATTTC	CTTCACAACCTCGTCGGCTA
Acaj-71920	Apoptosis inhibitor 5	TTTGCGTCAAACCTCTCTGC	GGAAGCTGTACATGAGGCA
Acaj-59320	Apoptosis regulator of BCL-2 family	CGCTAGGGTTGTTCGTCGA	GGAGAGCCAGTAGTAGAGC
Acaj-73477	Apoptosis regulator of BCL-2 family	GCGTCGAAAAGAACTGGTCA	CGTTCTTGAAGATGGGCCAG

*Oligonucleotides coupled to the T7 tail (italic) used for dsRNA synthesis.
