# Supplementary material for: The survival of Amblyomma sculptum ticks upon blood-feeding depends on the expression of an inhibitor of apoptosis protein
Source: Parasit Vectors. 2023 Mar 10;16:96. doi: 10.1186/s13071-023-05701-8 (PMC10007823; doi:10.1186/s13071-023-05701-8)

Additional file 3: Figure S1. A. Multiple sequence alignment of amino acid sequences was performed using MUSCLE method. BIR domains: grey boxes; RING domain: bold letters. **B.** Phylogenetic tree of tick IAPs. The tree was constructed with IAP amino acid sequences from 15 tick species (accession numbers available in Additional file 1: Table S1) using Maximum Likelihood (ML) method. Bar scale at the bottom indicates 10% amino acid divergence.

A.

```
Dermacentor reticulatus       ----------------------------EQTTVPDGPVVAKF----PKMAVALD-------YSREEDRLATFRG-WPPDAPVSAKKLAQG
Hyalomma excavatum            ------------MVRAELAQRGPPTLSGIEQSVVDGPPVSKF----SKMTNTATD------LSQEDKRLATFRE-WPPDAPVSAKKLAQG
Rhipicephalus pulchellus      ------------MMGTRLVQQGPHAFQAPEQTVLDGPMGAMVGKL-PKMANTTD-------YSREDVRLATFSG-WPQNAPVPAKKLAQG
Rhipicephalus appendiculatus  -----MCSMASTMVGTALAQRGPLAFRAPEQTVTDGPMGAAV----TKFTKMADT-AD---YNREQDRLNTFRG-WPPNAPVSAKKLAQG
Rhipicephalus zambeziensis    -----------MMVGTALAQPGPHAFRAHEQTVTDGPMGAAL----PKFAKMTTD------YSQEQVRHDTFRG-WPLNAPVSAKKLAQG
Rhipicephalus microplus       ------------------------------------------------------------------------RG----------------
Amblyomma maculatum           IRPRDRRQYFAISSGALLSFAHTVGNVHQNRHFLYGPLCSVMQW--HCFTVYASF------TSHAQDKLPGLHS-FSNDSNFR----IEG
Amblyomma triste              ---------MVLTMAERLAQRHPFP-PAREQTVTDGPLGSKL----VTSMATTTANRTVDLLSQEEVRLASFRG-WPLDAPVSAKKLAQG
Amblyomma sculptum            ----------------------------PEQTVTDGPLGVKF----TNMAAITRN-PD---LSQEDVRVASFRG-WPQSAPIPAKRLAQG
Amblyomma aureolatum          -----------------------------ERTVTDGPLGSK-----PISMDAVNMSPD---LSQEEVRLETFNNRWPPNPPISAKKLAQG
Ixodes ricinus                --------------MLEMGQMAFTHRRPEVTSVVDGPTNSHLKMAAPAVATSSGAAPSSPDLTQEEHRRRTFDS-WPQVSPTMALKLARA
Ixodes scapularis             --------------MLEMGQMAFTHHRPEVTSVVDGPTNSHLKMAAPAVATASGAAPSSLDLTQEEHRRRTFDS-WPQVSPTMALKLARA
Ornithodoros moubata          ------------------------------------------------------------------------------------------
Ornithodoros erraticus        MLKTISAAPLLGRNGDRMGSSTVDGGNQAKRALTPAHVGAQM-------------------LEEEQQRFETFGT-WPPDAPIAARKLARA
Ornithodoros turicata         ------------------------------------------------------------------------------------------


Dermacentor reticulatus       GFVCVSSPRRLTQCFKCGLEVSDWKIADSVVDRHRTVRPNCAFVRSLPSPRVPVAARPASSTSGASSVGTRRVEQPMHVDSVMPPLPRVG
Hyalomma excavatum            GFVCVSHTELVTKCFQCGVTVSNWKIADSVVDRHRTERPNCAFVRSLPSPRVSAAASPTSPTGVTSSGTARHPE-IMHVDSVVPPLPHTA
Rhipicephalus pulchellus      GFVCRDASGLVTQCFSCGLVVSDWSIADVVVDKHRTLRPGCAFVRSLPSPRISGAASPQSLTAATLSADARHVE-TMHVDHVAPPLPRMS
Rhipicephalus appendiculatus  GFVCKDASRLLTRCHSCKLEVSDWSIADVVVEKHRSMRPDCAFVRSLPSPRVSEAASPQSPTTATSSAGARHIE-RMDVDHVASPLPRMN
Rhipicephalus zambeziensis    GFVCRDASRLVTRCYSCGLEVSDWSIADVVVEKHRRMRPSCAFVRSLPSPRVSEAASPQSPTTATSSAGARLEE-RMQVDHVAPPLPRMN
Rhipicephalus microplus       ------------------------------------------------------------------------------------------
Amblyomma maculatum           GFVY-TGPGYRTRCFQCGVEIADWKIADSVVGRHRQARPECAFVCSLPAPTSAVQSPITSATTPPSFDTTRHPN-PVQADCVAQ--PRNA
Amblyomma triste              GFVY-TGPGYRTRCFQCGVEIADWKIADSVVGRHRQARPECAFVCSLPAPTSAVQSPITSATTPPSFDTTRHPN-PVQADCVAQ--PRNA
Amblyomma sculptum            GFVY-AGHGYLTRCFQCGLEIGDWKIADEVVKRHRQARPDCPFVRSLPSPASAALSPVSSTTTPPSFDNTRHST-PMQVDCLTQ--PRVG
Amblyomma aureolatum          GFVY-TGRGYHTRCFRCGLEITDWKIADSVVGRHRQARPNCTFVCSLPAPASAAQSPVSSATTPPLFSNS------TQVDCLTQ--PRVA
Ixodes ricinus                GFYH-VGRG-RTRCFSCGTECGDWRETQGAVERHRTLSPDCAFLRSVLGRSPPEP-----------------------------------
Ixodes scapularis             GFYH-VGRG-RTRCFSCGTECGDWRETQGAVERHRTLSPDCAFLRSMLGRSTSEP-----------------------------------
Ornithodoros moubata          ------------------------------------------------------------------------------------------
Ornithodoros erraticus        GFYY-TGQGLNTKCYECGIEHSGWVFSDSVMGKHKELSPNCEFVRKTFDGRSGECSASARRTSEP-------------------------
Ornithodoros turicata         --------------------------SDSAMGKHRELSPNCEFVLRSLGESSGGCSAGARRTSEP-------------------------


Dermacentor reticulatus       SAPLVRSSACEPVPGSA-VENDLQLRRLQASEEERFNTFSDWPLDFLPARTLAQAGFYYLHEQDKVRCAFCKGTVHNWEPGDDPVQEHAR
Hyalomma excavatum            SAPLVNSRFCEPVPRTG-VENGLVLQRLQASEEERFNTFYDWPLDYLPARALAQAGFYYIHEQDKVKCAFCKGVVHNWEPGDDPLREHAR
Rhipicephalus pulchellus      SAPSVQSRTCDTVPQSG-ARNDAPLHRLQASEEERFNTFYDWPLSSPSPRALAQAGFYYIHEQDKVKCAFCKGIVHNWEAGDDPLREHAR
Rhipicephalus appendiculatus  SAPSVQSRTCETVPQGG-ARNDVPLHRLQASEEERLNTFYDWPLTSPTPRALAQAGFYYIHEQDKVKCAFCKGVVHNWEQGDDPLREHAR
Rhipicephalus zambeziensis    SAPSVQSRTCETVPQGG-ARNDVPLHRLQASEEERVNTFIDWPLTSPTPRALAQAGFYYTHEQDRVKCAFCKGVVHNWEPGDDPLREHAR
Rhipicephalus microplus       -------------------------------------TFF--------------------------------------------------
Amblyomma maculatum           TVSS----FRVPAPSQIYKENDLLLRRLQASVEERINTFFDWPLDYISPRSMAEAGFYYLHEQDKVRCAFCRGTVHNWERGDDPLREHAQ
Amblyomma triste              TVSS----FRVPAPSQIYKENDLLLRRLQASVEERINTFFDWPLDYISPRSMAEAGFYYLHELDKVRCAFCRGTVHNWERGDDPLREHAQ
Amblyomma sculptum            SAPG-----VLRTLSQGFKENDLLLHRLQASEEERFNTFSDWPLDYLSPRLLAQAGFYYLHEQDKVRCAFCRGTVHNWERGDDPLHEHAR
Amblyomma aureolatum          TAPG----ACAPVSSQGFKENDLLLRRLQASEEERFNTFSDWPLDHLSPRLLAQAGFYYLHEQDKVRCAFCRGTVHNWERGDDPLREHAR
Ixodes ricinus                --------------------DEALLKR---SAAHRLRTFARWPLDFLDPTELARAGFYYLQQDDRVRCAFCRGTIHNWERGDDPLVEHGR
Ixodes scapularis             --------------------DEALLKR---SAAHRLRTFARWPLDFLDPTDLARAGFYYLQQDDRVRCAFCRGTIHNWERGDDPLVEHGR
Ornithodoros moubata          ------------------------MQR---SEQARLETFSSWPLDFIQPRELARAGFYYLEEGDRVRCAFCQGVIHNWEVGDVPLREHGR
Ornithodoros erraticus        --------------------DLAVMRR---SEDARLQTFANWPLDFLQPRDLARAGFYYLQQDDMVRCAFCHGVIHNWEPGDVPLREHGR
Ornithodoros turicata         --------------------DLALMQR---SEDARLQTFANWPVDFLQPRELAQAGFYYLQQDDMVRCAFCQGVIHNWERGEVPLREHGR
                                                                   **
```

```
Dermacentor reticulatus         HFPCCPFLLNPDL-AGQDECGHM-SWQRARSAPECEQLLVG-HGSQGVQLKGDTSPSALSGLGVSVHVGPKHPSQASPDARLRTFEKWPT
Hyalomma excavatum              HYPCCPFLLNPDL-AGHDECGHM-SWQQARSGPECAQLLVG-QGSQGVQLKGSTPPSELSGLGVSVHVGPKHPAQASPDARLRTFEKWPA
Rhipicephalus pulchellus        HYPCCRYLLNPDL-AGQDVCGHM-SWQQARSAPECAQLFIG-ESPPGVQLKGNTPPSELSGLGVSVHVGPKHPSQASPDARLRTYEKWPT
Rhipicephalus appendiculatus    HYPCCRYLLNPDL-AGQDVCGRM-SWQQERSAPECAQLFIN-ESPQGVQLKGNTPPSELYGLGVSVHVGPKHPSQASPDARLRTYEKWPT
Rhipicephalus zambeziensis      HYPCCRYLLNPDL-AGQDVCGRM-SWQQERSAPECAQLFIN-ESPEGVQLKGNTPPSELYGLGVSRHVGPKHPSQASPDARMRTYEKWPT
Rhipicephalus microplus         ------------------------------------------------------------------------------------------
Amblyomma maculatum             HYPCCPFLLDPSL-AGQDECGHE-SFHRARSVPEDRHLLIGANGSQGVQLKGGTPPSSLSGLGVSMHVGPKHPSQASPDARLRTFEKWPT
Amblyomma triste                HYPCCPFLLDPSL-AGQDECGHE-SFHRARSVPEDRHLLIGANGSQGVQLKGGTPPSSLSGLGVSMHVGPKHPSQASPDARLRTFEKWPT
Amblyomma sculptum              HYPCCPFLLDPSL-AGQDECGRE-SWHRSRSVPEGRHLLIGANGTQGVQLKGDTPPSELSGLGVSVHVGPKHPSQASPDARLRTFEKWPS
Amblyomma aureolatum            HYPCCPFLLDPSL-AGHDECGHE-SWHRSRSVPEGRQLLIGANGSQGVQLKGDTPPSELTGLGVSMHVGPKHPSQASPDARLRTFEKWPT
Ixodes ricinus                  HFPCCPFLLDPSLEEGQDECGR--SWREQRSHSE--------RGAAPPVEGGHSADSPLSLLGITPHAGPKHPAQASPDARLRSFAKWPP
Ixodes scapularis               HFPCCPFLLDPSLEEGQDECGH--SWREQRSHSE--------RGPAPPVEGGHSADSPLSLLGITPHAGPKHPAQASPDARLRSFAKWPP
Ornithodoros moubata            HFPCCPFLLDPSLTATSDVCGRFNSFHETLSGPE--------KTSIGSK---DSVHAPLAKLGIALHSAPKHSSLVSPSARLRSFEQWPA
Ornithodoros erraticus          HFPCCPFLLHPSVEPSYDVCGPFSSFHERLSGPE-----------KKTNIAERSHPTPLTRLGIALHSAPKHTALVSPDARLRSFEAWPA
Ornithodoros turicata           HFPCCPFLLNPSMRSSMDVCGRFSSFNEVLSGPEKRAI-----------ASQDSAHVPLTRFGIAPHSAPKHTALVSPDARLRSFEEWPS

Dermacentor reticulatus         TCPKQPQELVPAGFFYIGLQDYTKCFHCDGGLCNWDATDDPWEEHARWFPRCQFVLLSKGETYVEDCLRRHQSHLSAVAASASTSAQGQS
Hyalomma excavatum              TCAKRPQELIVAGFFYIGVQDFTKCFHCDGGLCNWDANDDPWEEHARWFPRCQFVLLSKGEAYVKDCLRRHQSHLSAVAASASTSSQGQS
Rhipicephalus pulchellus        TCPKRPQELVVAGFFYIGVNDYTKCFHCDGGLCNWDSNDDPWEEHARWFPRCQFVLLSKGEAYVQECLKRHQSHLSAVAASASTSSQGQS
Rhipicephalus appendiculatus    TCPKRPQELVVAGFFYIGVNDYTKCFHCDGGLCNWDSNDDPWEEHARWFPRCQFVLLSKGETYVQDCLKRHQSHLNAVAASASTSSQGQS
Rhipicephalus zambeziensis      TCPKRPQELVVAGFFYIGVNDYTKCFHCDGGLCNWDSNDDPWEEHARWFPRCQFVLLSKGETYVQECLKRHQSHLSAVAASASTSSQGQS
Rhipicephalus microplus         -------------FLFIGVNDYTKRFHCDGGLCNWDVNDDPWEEHARWFPRCQFVLLSKGESYVQDCLKRHQSHLSAVAASASTSSQGQS
Amblyomma maculatum             TCPKRPSELVQAGFFYIGVQDYTKCFHCDGGLCNWDTGDDPWEEHARWFPRCQFVLLAKGEAYVQDCLRRHQSHLSSVAAS--SSQQGQT
Amblyomma triste                TCPKRPSELVQAGFFYIGVQDYTKC-----------------------------------------------------------------
Amblyomma sculptum              TCVKRPLELVQAGFFYIGVQDYTKCFHCDGGLCNWDSGDDPWEEHARWFPRCQFVLLTKGEAYVEDCLRRHQSHLNSVAAS--SSHQGQT
Amblyomma aureolatum            TCAKRPLELVQAGFFYIGVQDYTKCFHCDGGLCNWDTGDDPWEEHARWFPRCQFVLLAKGEAYIQDCLRRHQSHLNSVAAS-TSSQQGQT
Ixodes ricinus                  ASPLRPPDLVKAGFFYIGILDYTKCFHCDGGLCNWERGDDPWEEHARWFPKCQFVLLSKGDAFVRDSVQKHLEHMSQVARS--GSAALAE
Ixodes scapularis               ASPLRPPDLVKAGFFYIGILDYTKCFHCDGGLCNWERGDDPWEEHARWFPKCQFVQITKGDTLGRDTRQKQLQNTRKTART--ASAALAE
Ornithodoros moubata            GVPAKPEDLVKAGFFYIGLNDYTKCFHCDGGLCNWEAGDDPWVEHARWFPNCEFVRLSKGDDFVKECADKQKSHFEAIATALPQCSK---
Ornithodoros erraticus          DAPIRPQDLIKAGFFYIGLNDYTKCFHCDGGLCNWEAGDDPWVEHARWFPGCEFVRLTKGEAFIKECADKQKTHFEQIATTLPQTCE---
Ornithodoros turicata           DAPVRPHDLVKAGFFYIGLNDYTKCFHCDGGLCNWETGDDPWVEHARWFPGCEFVRLNKGVAFIKECSDKQKSHFQAIATTLPQSSE---
                                             *::**: *:**

Dermacentor reticulatus         GGADEGMATELAALMRSEDVQFYLSQGVPAETLRAALLKHMRCQGRGFASRDELLQVLGELLALPRASADQTPQPSAANGLTITKLATNP
Hyalomma excavatum              GGEDEGMATELAALMRSEDVQFYLSQGVPAETLRAALLKHMRSQNCGFASRDELLQVLGELLALPRASADQTPQRSATNGVAVTKVATSP
Rhipicephalus pulchellus        GGADEGMATELAALMRSEDVQFYLSQGVPAETLRAALLKHMRSQGHGFASRDELLQVLGELLTLPRSSADQSPQRSATNSVTITKVATNP
Rhipicephalus appendiculatus    GGADEGMATELAALMRSEDVQFYLSQGVPAETLRAALLKHMRSQGRGFASRDELLQVLGELLTLPRASADQSPQRSATKSVTITKVATDP
Rhipicephalus zambeziensis      GGADEGMATELAALMRSEDVQFYLSQGVPAETLRAALLKHMRSQGRGFASRDELLQVLGELLTLPRASADQSPQHSATNSVAITKVATDP
Rhipicephalus microplus         GGAEDGMTTELAALMRSEDVQFYLSQGVPAQTLRAALLKHMRSQNRGFASRDELLQVLGELLTLPRASADQSPQRSATNSVTITKVATNP
Amblyomma maculatum             EAADEGMATELAALMRSDDVQFYLSQGVPAETLRAALLRHMRGQGRGFASRDELLQVLGELLALPRASADQTLQGQAANDA---------
Amblyomma triste                ------------------------------------------------------------------------------------------
Amblyomma sculptum              EAADEGMATELAALMRSDDVQFYLSQGVPAETLRAALLQHMRGQGRGFASRDELLQVLGELLALPKSSADQTPLEQAANGA---------
Amblyomma aureolatum            EAADEGMTTELAALMRSDDVQFYLSQGVPAGTLRAALLQHMRGQGRGFASRDELLQVLGNLLALPRASADQTPQEQTASGA---------
Ixodes ricinus                  HGAQPGMETEVAALLRSGDVQFYVDQGVDRETLRVALLAHVQRQQRGFSSRDELIQVLASLFSLHRRQPEAAVSSTADFHQPSSGAAELA
Ixodes scapularis               HGAQPGMETEVAALLRSGDVQFYVDQGVDRETLRVALLAHVQRQQRGFSSRDELIQVLASLFSLHRRQPEAAVSSTADFHQPSSGAAEPA
Ornithodoros moubata            ---EPNVESELNTLMQSPDVLFYAEQGVPEDTLRKSLRAHMVHTGRGFSSREELTEVLNSMFSLNATRLENLANHAEDEVLKVVRK----
Ornithodoros erraticus          ---DTNIESELNTLMQSGDVKFYMEQGVPQNTLRKSLGAHIARTGRGFSSREELTQVLNSMFSLNAARSANSSVSTEGGSLPAKGTGDDG
Ornithodoros turicata           ---DPNIESEMDTLMQSPDVLFYVEQGVPLDILRNSLRAHIASTGRGFSSREELTQVLNSMFSLNTARLGDPSNSKESRGSPAKATAD--
```

Dermacentor reticulatus	-QLETTVSRAKNSSPEGRMEGSEPTELALENL**RLKDQRLCKVCLDAEVGVVFLPCGHLVACPACASALSDCPVCRASIRGTVRTFFS**
Hyalomma excavatum	EQKESLLSRAKNSLSEGLSEGSEPSDLALENL**RLKDQRLCKVCLDAEVGVVFLPCGHLVACPACASALSDCPVCRASIRGTVRTFFS**
Rhipicephalus pulchellus	-ETEGVVSRAKNSSSESLTEGSESSDLALENL**RLKDQRLCKVCLDAEVGVVFLPCGHLVACPACAAALSDCPVCRAAIRGTVRTFFS**
Rhipicephalus appendiculatus	-HTEGVVSRAKNASSESLTEGSESSDLAMENL**RLKDQRLCKVCLDAEVGVVFLPCGHLVACPACASALSDCPVCRAAIRGTVRTFFS**
Rhipicephalus zambeziensis	-HTEGVVSRAKNASSESLTEGSESSDLAMENL**RLKDQRLCKVCLDAEVGVVFLPCGHLVACPACASALSDCPVCRASIRGTVRTFFS**
Rhipicephalus microplus	-QTEGIVSGAKNASSESLTEGLESSDLALENL**RLKDQRLCKVCLDAEVGVVFLPCGHLVACPACASALSDCPVCRASIRGTVRTFFS**
Amblyomma maculatum	-ANKNIFSDAGKSLSPTVKMAAVSKSSDPEVT**RLNDQRLCKVCLDAEVGVVFLPCGHLVACPSCASALVDCPICRASIKGTVRTFFA**
Amblyomma triste	---
Amblyomma sculptum	-SSKNIFSAAGKQSSPAVVTPVESEPSDPEHT**RLKDQRLCKVCLDAEVGVVFLPCGHLVACPSCASALVDCPICRAAIRGTVRTFFA**
Amblyomma aureolatum	-SAKSIFSGAEKSSLAAVLTPAGSEPSDPEDT**RLKDQRLCKVCLDAEVGVVFLPCGHLVACPSCASALVDCPICRAPIRGTVRTFFA**
Ixodes ricinus	SEQHAAKEEPPRTPLTAEGPAHDPEELQLENL**RLKEQRLCKICLDAEVGVVFLPCGHLVACPACAASIKDCPVCRKTIVGAVRTFLS**
Ixodes scapularis	SEQPAAKEEPPRTPQSAEGPAHDPEELQLENL**RLKEQRLCKICLDAEVGVVFLPCGHLVACPACAASIKDCPVCRKTIVGAVRTFLS**
Ornithodoros moubata	-KHAAPVPCDKQQSSHTSGGASEAERLELENL**RLKEQRLCKICLDAEVGIVFLPCGHFAACPACAASLKDCPICRKPIMGSVRTFLA**
Ornithodoros erraticus	SEQP---SNSVSISTHTGGGVSGTELLELENL**RLKEQRLCKICLDAEVGVVFLPCGHLVACPACGSSLKDCPICRKTIMGNVRTFLS**
Ornithodoros turicata	-KNTVQIQCDEQRNGRTGGGASDTERLELENL**RLKDERLCKICLDAEVGVVFLPCGHLVACPACAASVKDCPICRKTIMGSVRTFLS**

B.

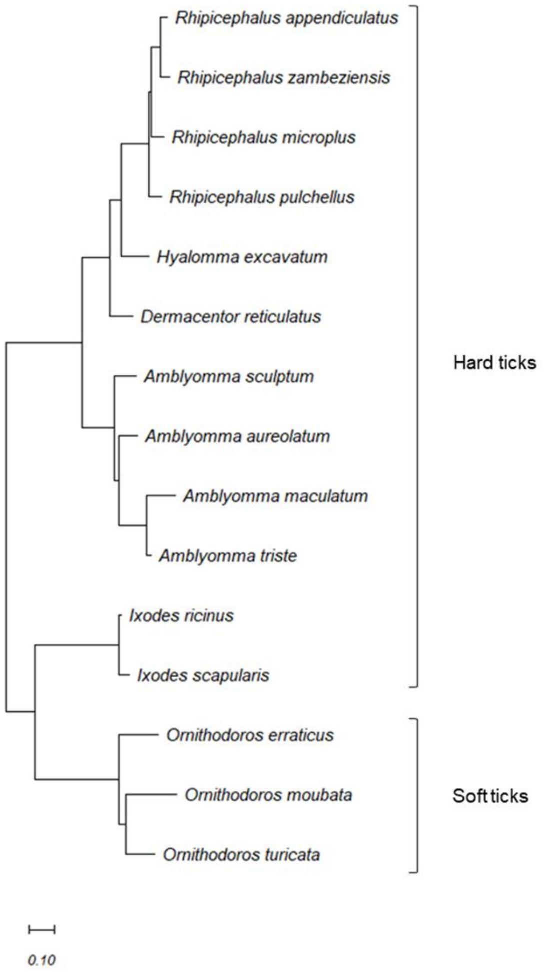

Supplement: Supplementary file 3 — Additional file 3: Figure S1. A Multiple sequence alignment of amino acid sequences was performed using the MUSCLE method. BIR domains are shown in gray boxes, and RING domain is shown in bold letters. B Phylogenetic tree of tick IAPs. The tree was constructed with IAP amino acid sequences from 15 tick species (accession numbers available in Additional file 1: Table S1) using the maximum likelihood method. The bar scale at the bottom indicates 10% amino acid divergence. [file 13071_2023_5701_MOESM3_ESM.pdf]
