# Supplementary figures and images for: The survival of Amblyomma sculptum ticks upon blood-feeding depends on the expression of an inhibitor of apoptosis protein
Source: Parasit Vectors. 2023 Mar 10;16:96. doi: 10.1186/s13071-023-05701-8 (PMC10007823; doi:10.1186/s13071-023-05701-8)

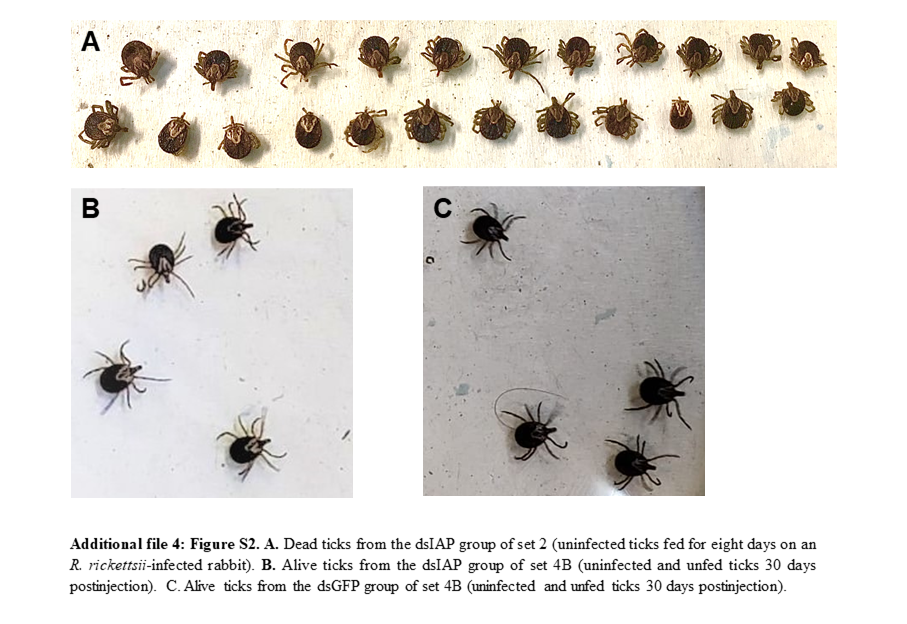

Supplement: Supplementary file 4 — Additional file 4: Figure S2. A Dead ticks from the dsIAP group of set 2 (uninfected ticks fed for 8 days on an R. rickettsii-infected rabbit). B Live ticks from the dsIAP group of set 4B (uninfected and unfed ticks 30 days postinjection). C Live ticks from the dsGFP group of set 4B (uninfected and unfed ticks 30 days postinjection). [file 13071_2023_5701_MOESM4_ESM.tif]
